# The impact of COVID-19 safety interventions on creating a controlled environment on campus

**DOI:** 10.3389/fpubh.2022.962478

**Published:** 2022-09-23

**Authors:** Sana Mahmood, Sonia Ijaz Haider, Hamna Shahbaz, Ali Aahil Noorali, Noreen Afzal, Aziz Jiwani, Samar Zaki, Unab Iqbal Khan, Khairulnissa Ajani, Muhammad Tariq, Rozina Karmaliani, Adil Hussain Haider

**Affiliations:** ^1^Aga Khan University, Karachi, Pakistan; ^2^Centre for Medical Education, School of Medicine, Cardiff University, Cardiff, United Kingdom; ^3^Department of Surgery, Washington University in St. Louis, St. Louis, MO, United States; ^4^Department of Medicine, Aga Khan University, Karachi, Pakistan; ^5^Department of Family Medicine, Aga Khan University, Karachi, Pakistan; ^6^School of Nursing and Midwifery, Aga Khan University, Karachi, Pakistan

**Keywords:** feedback, medical education research, public health, COVID-19, mixed-methods study, medical students, nursing-education

## Abstract

**Objectives:**

During COVID-19 the re-opening of educational institutes was frequently debated, however with the decline in the number of COVID-19 cases, The Aga Khan University (AKU) in Karachi, Pakistan opened its campus for medical and nursing students after more than 6 months of closure. To ensure gradual resumption of activities on-campus, a combination of interventions was diligently deployed to minimize student infection rates. Scarce literature exists on students' perceptions regarding decisions implemented by university leadership. The aim of the study was to determine the efficacy of these interventions.

**Methods:**

We conducted a convergent, parallel, mixed-methods observational study targeting medical and nursing students. An online questionnaire was disseminated to elicit students' degree of (dis)agreement on a four-point Likert scale. Focused group discussions (FGDs) were conducted to comprehend reasons for (dis)agreement.

**Results:**

Total of 183 students responded to questionnaire (59.0% nursing, 67.8% female), 11 FGDs were conducted with 85 students. Interventions with highest agreement were mandatory face masks policy (94.54%), weekly mandated COVID-testing (92.35%) and students' Academic Bubble (91.26%); highest disagreement was for Sehat Check application (41.53%); and stay strong campaign (40.44%). Four themes emerged from FGDs: Effective safety interventions, Safety interventions with limited effectiveness, Utility of Sehat Check Application and Future recommendations for informing policy.

**Conclusion:**

It is paramount to seek student-feedback at forefront of university re-opening strategy. Clear communication channels are as important as an administrative response system's robustness. Bidirectional communication channels are fundamental and requisite during ever-changing policies and regulations. Engaging student representatives in decision making or implementation processes (such as “pilot” before “roll-out”) would allow any potential issues to be managed early on. Gather real-time anonymous feedback and identify key areas that need further promulgation and those that need to be replaced with more effective ones.

## Introduction

The COVID-19 pandemic has cut a swath globally, resulting in an unprecedented disruption in social, economic and educational systems. The rapid evolution of the pandemic dictated that critical decisions be made regarding the closure of educational institutions to curb the spread of disease and put a halt to soaring infection rates (1, 2). While clinical clerkships and in-person educational activities are of paramount importance to the practical learning of medical students, the Association of American Medical Colleges (AAMC) released guidelines for the immediate suspension of clinical activities on-campus in medical schools across United States (3). A similar trend also followed worldwide, with increasing concerns regarding the quality of education and practical training received by medical students, the attainment of profound clinical skills and the “imposter syndrome” (4–6). Consequently, traditional training and teaching methods have been gradually replaced by synchronous and asynchronous virtual modalities (5, 7, 8).

With the global trends of disease spiraling, one of the most contentious topics has been the re-opening of universities and campuses, while adequately ensuring effective safety measures for students returning back to campus vicinities (9–11). Since a one-size-fits-all approach cannot be effectively utilized in a global context, most educational institutions have employed a multi-pronged approach, leveraging a variety of public health strategies for reducing infection rates on campus (12).

On February 26, 2020, the first positive case of coronavirus disease (COVID-19) was identified in Pakistan (13). To counter the spread effectively, the government of Pakistan shut down all educational institutions, mosques, and leisure areas (14). All meetings and services were postponed, marriage halls closed and sporting events canceled. A full lockdown was enforced in the country on March 23, 2020 (14).

Other preventive measures included were travel restrictions, quarantine shelters, cordoning off areas, testing and contact tracing, implementation of mask, sanitizers, and social distancing, awareness campaigns, and production of ventilators (15).

With the decline in the number of COVID-19 cases in September 2020, the Federal Ministry announced a phased approach of opening educational institutions, depending on the evolving situation (16). The Aga Khan University (AKU) in Karachi, Pakistan also opened its campus for medical and nursing students after more than 6 months of closure. To ensure gradual resumption of activities on-campus, a combination of interventions was diligently deployed by the COVID-19 Command Center at AKU to minimize student infection rates. While studies (17–19) have reported data on the efficacy of such interventions, there is a dearth of literature on the perspectives of key stakeholders impacted by these policies—students themselves. All interventions impact students either positively or negatively, and therefore their perceptions are important to ensure maximum support is provided to facilitate them in their academic endeavors (20, 21).

Therefore, the aim of this present study was to explore students' perceptions around these interventions: Specifically, the research objectives were:

1. To determine the usefulness of the interventions in creating a safe and controlled environment on campus (systems-level feedback).
2. To evaluate the ability of the intervention to influence students practice of safety measures and adherence to infection-control protocols (behavior change feedback).

## Methods

### Study design

We selected a convergent, parallel, mixed methods study design to explore students' perceptions and degree of agreement toward usefulness of interventions to control student infection rates on campus (22–24). Prior to commencement, we obtained ethical approval by the Institutional Review Board (ERC Reference No: 2020-5640-15085).

**Figure 1:**
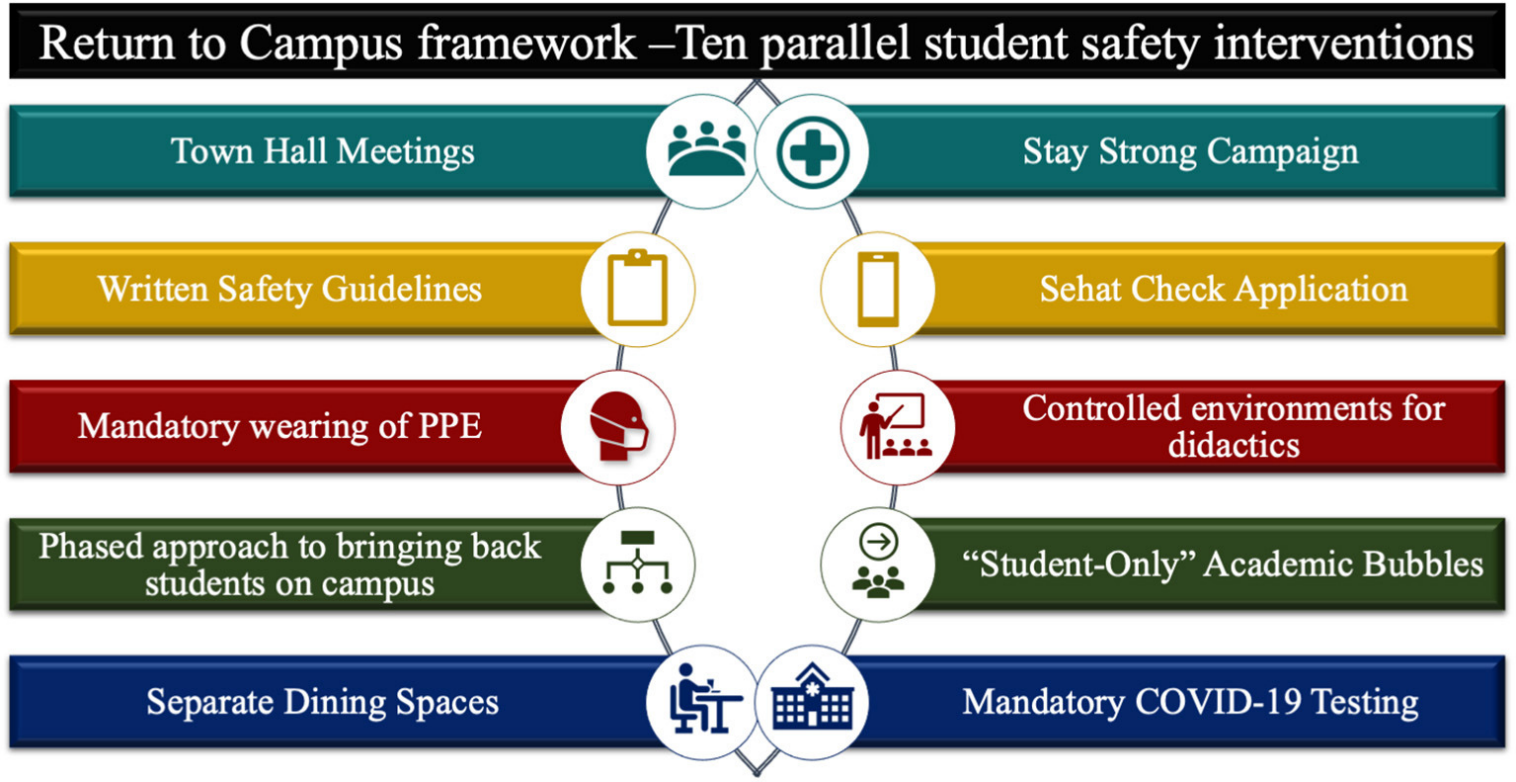
Ten parallel student safety interventions.

The organizational committee implemented a combination of 10 cross-cutting and concurrent interventions to create a safe and controlled environment (23) (Figure 1).

### Setting and participants

The Aga Khan University Hospital is a tertiary-care, teaching hospital centered in the metropolitan city of Karachi, Pakistan (25). It offers two flagship health sciences' programs amongst other academic programs. The 5-year medical school program, comprises of pre-clinical years (Years 1–2) focusing on basic sciences, and clinical years (Years 3–5), where students are immersed in discipline-based clinical clerkships (26). Similarly, the School of Nursing and Midwifery (SONAM) offers a Bachelor of Science in Nursing (BScN), a 4-year program with the last 2 years (Years 3–4) focusing on clinical rotations (27).

Our study enrolled students in clinical years both in medical school (group A) and nursing school (group B). These cohorts were selected because they returned to campus premises following commencement of classes in mid-September, in accordance with regulations of the Ministry of Education in Pakistan (16). Using convenience sampling, all students who volunteered to participate and signed the written informed consent form participated in the study.

### Interventions

The 10 interventions were developed by the COVID-19 Command Center at the Aga Khan University. This overarching governance body has representation from academic leadership, hospital and service leadership, infection prevention teams and student health teams. Student representation was integral to the development of these interventions and rigorous, continuous feedback on a regular basis. The Student Taskforce Against COVID-19 (STAC-19), a student volunteer taskforce, routinely interacted with the Command Center leadership for discussions around strategy, implementation, and feedback of these interventions (28).

The interventions can be divided into four major categories: 1. Communications and student wellbeing, 2. University strategy and hospital policy, 3. Screening application “Sehat Check” and 4. Protective measures and Testing.

#### Communications and student wellbeing

To ensure students' queries were aptly conveyed and acted upon, the leadership regularly met students *via* town-hall meetings (online/in-person). Written safety guidelines and pictorials were disseminated through emails and displayed in clinical areas and student spaces. The “Stay Strong” campaign targeted students' wellbeing by encouraging them to follow safety guidelines (*via* social media, socially distant gatherings, and university communications -brochures and posters) and providing access to important contact information.

#### University strategy and hospital policy

Every study space and academic teaching room had “Maximum Capacity” posters displayed on entrance doors to limit the number of students during didactic in-person sessions. An “Academic Bubble” was created, by restricting certain areas as student-only zones, prohibiting access to visitors and patients. A separate dining space was arranged for students (previously combined with the hospital dining space). Students returned in a staggered manner, starting with senior clinical years, followed by junior years (Year 5, then Year 4, then Year 3) with a gap of 2 weeks between consecutive batches.

#### Sehat check application

A novel mobile application “Sehat Check App” (Sehat = Urdu word for Health) was launched which deemed it necessary for students to screen themselves (symptom-based) regularly each morning to enter the university (29). It consisted of brief screening questions (e.g., presence of cough, fever, body aches etc. or exposure to a person with COVID). Answering “yes” to any one of the screening questions resulted in the application showing a red mark, indicating the need for further assessment and guided the user for testing purposes.

#### Protective measures and testing

Students were mandated to wear personal protective equipment from day one. Mandatory wearing of masks was instituted in all areas of the university and hospital. However, certain higher-risk areas such as the Emergency Room and the Endoscopy Suite, also required the additional wearing of face-shields and gowns. New face masks and face shields were biweekly distributed. Weekly mandatory COVID-19 testing was instituted (each Monday morning) to identify asymptomatic cases early on.

### Quantitative component

A structured questionnaire was developed to elicit students' perceptions about the usefulness of interventions. In November and December 2020, an online questionnaire was disseminated to all students (*n* = 557), using a secure link provided *via* email. The first part of questionnaire enquired demographic details of students, level of exposure to other people based on time spent on campus, number of individuals in their household and mode of transportation. The second part of questionnaire, split into two parts consisting of 10-items each, focused on assessing the effectiveness of interventions. Students had to rate their degree of (dis)agreement on a four-point Likert Scale. All questionnaires were pilot tested, anonymized with de-identified codes assigned to each participant to maintain confidentiality.

### Qualitative component

Students were invited *via* email to participate in focused group discussions (FGDs) to comprehend reasons for effectiveness of interventions and explore further recommended strategies. Participation in FGDs was voluntary and confidentiality of data was maintained by assigning unique de-identified codes to each participant. A semi-structured interview guide was developed, pilot tested, and six trained interviewers discussed its final version to ensure standardization. Each session spanned an hour and included between 8 and 12 students. Eleven FGDs were conducted, involving 54 medical and 31 nursing students. Data collection was completed upon thematic saturation.

### Data analysis

#### Quantitative analysis

Data from quantitative surveys were analyzed using StataCorp. 2019 (Stata Statistical Software: Release 16. College Station, TX: StataCorp LLC). Frequency and percentages were used to calculate the demographic characteristics and variables.

#### Qualitative analysis

Initially, the audio recordings from the students' discussions were transcribed. In the transcriptions, no identifying features/characteristics were included. Colaizzi's analysis method (30) was used to analyze the transcript. The analysis included steps such as familiarization, identifying significant statements, formulating meanings, clustering themes, developing an exhaustive description, producing the fundamental structure, and seeking verification of the fundamental structure. This was done through an iterative process where participants' data were coded, compared, contrasted, and refined to develop emergent themes. The transcribed text was separated into “meaningful units” which were further shortened and labeled with a “code.” Codes were then analyzed and assembled into similar categories. In the last step, comparable categories were grouped under subthemes and main themes. Two researchers were involved in independently reviewing the data and formulating the themes after summarizing and extracting the meaningful contents, bracketing the presuppositions of the researchers (using QSR NVivo—version 12, Melbourne, Australia). Any inconsistencies were solved through discussion until a mutual agreement was reached.

## Results

### Demographics

A total of 183 students completed the survey, of which 124 (67.8%) were females and 59 (32.2%) males. 108 (59.0%) nursing and 75 (41.0%) medical students participated. The highest number of students belonged to third year, 85 (46.4%), followed by fourth year 78 (42.6%). 102 (55.7%) of the students lived in campus dormitories and 81 (44.3%) lived off campus and commuted to the university daily. Of these 81 students, most students lived in households with 4–5 inhabitants 29 (35.8%) and 2–3 inhabitants 39 (48.15%). Further, 37 (45.7%) students used shared private transport and 21 (25.7%) used shared public transport to commute to campus (Table 1).

**Table 1:** Snapshot of study participants—demographics and exposure characteristics (*n* = 183).

**Variables**	**Frequency (*N*)**	**%**
**Demographic distribution**		
**Gender**		
Females	124	67.8
Males	59	32.2
**Age**		
20	10	5.5
21	40	21.9
22	66	36.1
23	44	24.0
24	17	9.3
Above 24	6	3.3
**Academic affiliation**		
SON (Nursing students)	108	59.0
MC (Medical students)	75	41.0
**Year of study**	85	
3rd year	78	46.4
4th year	19	42.6
5th year		10.4
**Residential status**		
Off campus	81	44.3
On campus hostel/dormitory	102	55.7
**Degree of exposure to other individuals**		
**Duration of stay on campus (weeks)**		
< 2 weeks	12	6.6
2–4 weeks	16	8.7
4–6 weeks	20	10.9
6–8 weeks	32	17.5
>8 weeks	103	56.3
**Area of preference while studying**		
Personal Room—Off Campus (closed individual space)	42	23.0
Personal Room—On Campus (closed individual space, shared dorms)	85	46.5
University Library (closed group space)	41	22.4
University Learning Resource Center (closed group space)	12	6.6
University Courtyard (open group space)	1	0.6
Others	2	1.1
**Area of preference while spending free time**		
Personal Room—Off Campus (closed individual space)	37	20.2
Personal Room—On Campus (closed individual space, shared dorms)	44	24.0
University Courtyard (open group space)	34	18.6
Sports Center (open group space with close contact)	46	25.1
Student Lounge (closed group space)	22	12.0
**Mode of transport to campus (off campus dwellers)**	***(n*** **= *81)***	
Private—Individual	23	28.4
Private—Shared (>1 person in car)	37	45.7
Public—Shared (>1 person in car)	21	25.9
**Number of habitants in household (off campus dwellers)**	***(n*** **=** ***81)***	
< 2	0	0.0
2–3	29	35.8
4–5	39	48.2
6–7	9	11.1
>8	4	4.9

A total of 85 students participated in 11 FGDs. Six were conducted at medical college and five at school of nursing. Within these FGDs, 54 medical students from third, fourth and fifth year along with 31 nursing students from third and fourth year participated in the study.

### Quantitative

**Figure 2:**
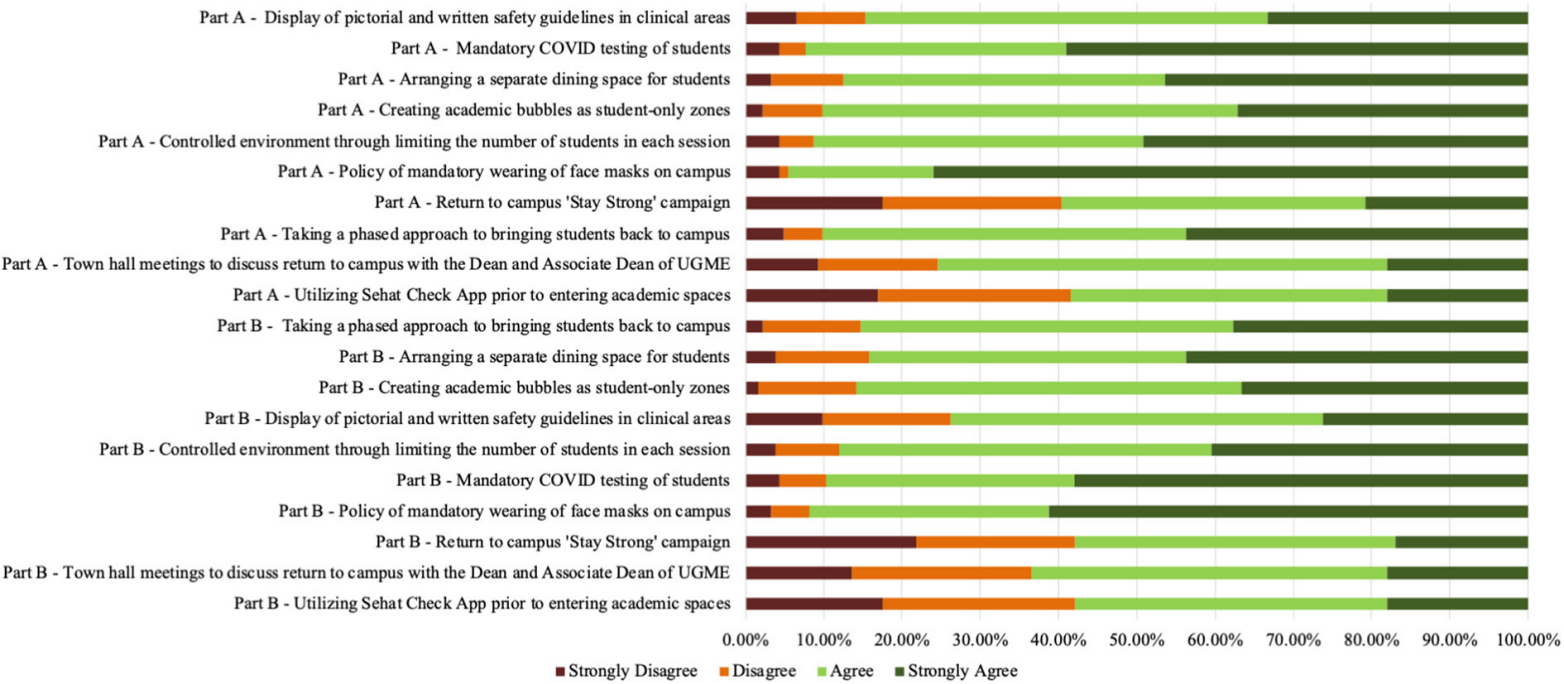
Students' degree of agreement/disagreement toward the efficacy of safety interventions.

The first part of survey asked students to assess the intervention's ability to create a safe environment for infection control. Significant agreement (strongly agree and agree) was recorded for the following interventions (Figure 2): policy of mandatory wearing of face masks on campus (94.54%), weekly mandatory COVID testing of students (92.35%), limiting number of students on campus (91.26%), using a phased approach to bring students back to campus (90.17%) and creating academic bubbles as student-only safe zones (90.17%). Students showed highest disagreement toward Sehat Check application (41.53%) and stay strong campaign (40.44%).

The second part of survey focused on the contribution of intervention to respondents' practice of safety measures and adherence to infection-control protocols. Similar results were observed, with highest agreement shown for the face mask policy (91.80%), weekly mandatory COVID testing of students (89.61%) and limiting number of students in each session (87.98%). Likewise, highest disagreement was observed for the Sehat Check application (42.08%) and the Stay Strong campaign (42.08%).

### Qualitative

Based on the data collection and thematic analysis, four overarching themes were identified. Table 2 shows the main themes along with their sub-themes and codes. Table 3 identifies pertinent quotations from students' verbatim.

**Table 2:** Thematic analysis of focused group discussions with themes, sub-themes, codes, and key messages.

**Themes**	**Subthemes**	**Code**	**Descriptors/Key messages**
1. Effective interventions	1.1 Group agreement toward the intervention	• A • B • C • D • E • F • G • H • I	• Mandatory COVID testing of students • Policy of mandatory wearing of face masks on campus • Controlled environment by limiting students in sessions • Creating academic bubbles as student-only zones • Arranging a separate dining space for students • Provision of Personal Protective Equipment • Phased approach to bringing students back to campus • Restricting student entry into ER and Special Care units • ^*^ University emphasis on Social Distancing
2. Ineffective interventions	2.1 Lack of group consensus toward the intervention	• A • B • C • D • E	• Display of pictorial and written safety guidelines • “Stay strong” campaign • Town hall meetings to discuss issues with return to campus • Restricting food delivery services • ^*^ Implementation of a curfew (11 p.m.) on campus
	2.2 Barriers to implementation of interventions	• A • B • C • D	• Decreased COVID-19 testing with progression of weeks • Academic Bubble—Instances of unauthorized access • Communication limitations—Messages regarding student SOPs not effectively disseminated to attendings • Online sessions—Lack of standardization across specialties
	2.3 Effects on Mental Health	• A • B	• Academic and interpersonal stressors • Anxiety
3. Sehat check application	3.1 Strengths	• A • B	• Utility as a screening tool • Effective in pushing to get tested
	3.2 Limitations	• A • B	• Allows for dishonesty since symptoms are self-reported • Redundant in asymptomatic patients
	3.3 Reasons for compliance	• A • B • C	• Entry Ticket • Exiting hostels • Entering dining areas
	3.4 Reasons for lack of compliance	• A • B • C • D • E	• Inconsistency of guards who check • Lack of time • Forgetting to fill it • Lack of internet access at certain points • Not experiencing any symptoms
	3.5 Recommendations for improvement	• A • B • C • D • E • F • G	• Adding a double check option before submitting response • Adding the option of contact tracing • Adding COVID-19 results in the app • Asking more comprehensive and specific questions • Making it functional without internet • Making COVID-19 consultation appointments • Making the response system efficient in case of symptoms
4. Future recommendations	4.1 Targeted at juniors yet to return to campus	• A • B • C	• Honestly reporting symptoms • Limiting unnecessary movement • Wearing masks and maintaining social distance
	4.2 Targeted at leadership that strategizes and implements university policy	• A • B • C • D • E	• Inclusion of students in decision-making • Regularity in COVID-19 testing • Body temperature checks at entry points • Immediately notifying about students/staff testing positive • Creating an anonymous student body communication channel for reporting exposure and breach of SOPs

* These items were not part of the 10 items in the “Return to Campus” portfolio, but were still mentioned by students as other interventions that they found effective/ineffective.

**Table 3:** Representative student quotations corresponding to themes and sub-themes.

**Themes**	**Sub-themes**	**Quotations**
1. Effective interventions	1.1 Group agreement toward the intervention	**Fourth Year, MBBS student, 23 y Male:** “Having our space is helpful because that restricts the patient influx through the medical college…” **Fourth Year, MBBS student, 23 y Male:** Second thing that was very effective was having our own space for lunch. As a day scholar, I just have lunch but now they have started dinner as well which is even better. Because we do not have to go to the main cafeteria and get exposed to people who are not tested. **Third Year, BSCN student, 21 y Female:** “because most of the people who were infected were (actually) asymptomatic so, I think the intervention of mandatory test is most effective in limiting spread of infection” **Fifth Year, MBBS student, 24 y Female:** “Most important measures, for me were, number one mandatory wearing of face masks, and it has been proven by research that it limits the spread of COVID-19 and also in all of the cases in which students tested positive in my batch, they didn't spread it further because they were wearing masks most of the time.” **Fifth Year, MBBS student, 24 y Male** “since we started with the mandatory testing and everybody tested negative and then we started our rotations, it really helped create a safer environment.” 4th Year, MBBS student, 23y Female, “The online sessions have also been very effective and limiting the number of people in one in person group has also been effective but online sessions are preferred over in person”
2. Ineffective interventions	2.1 Lack of group consensus toward the intervention	**(Restricting university spaces)** **Third Year, BScN Student, 21 y, Female:** “If university is closing the doors at 11 p.m. and we are not allowed to go outside I think they should not do this. Everyone should be allowed to go outside hostel in the campus because this is the place where we can study, and we can go to SRC and play and release our stress there. And these activities are very important because we are having hectic schedules and we have to balance everything. So do not close the doors and limit our movement.” **(Stay Strong campaign)** **Fourth Year, MBBS Student, 23y Female:** “Whereas I think there were a lot of commercial things such as the ‘stay strong campaign' which really did not do much. At least it did not have any longevity to it” **Fifth Year, MBBS student, 24 y Male:** So in my opinion, considering talking about rounds, I feel that the number of people who are involved in the rounds can definitely be reduced, because right now 10–12 people are rounding at the same time. So it's not possible to maintain social distance,
	2.2 Barriers to implementation of interventions	**Fourth Year, MBBS student, 24 y Male:** There have been a lot of times where we see random people just in the academic bubble who are not supposed to be there.” **Fourth Year, MBBS student, 24 y Female:** “We were given the patients in the special care wing. But we have been exclusively told, exclusively told not to go there and when I told the doctor on the rounds that we are not supposed to go here, she is like these are the only patients we have so you have to kind of go, “wear a mask, do this, go inside, there is no problem.” **Fourth Year, MBBS student, 24 y Female:** “I also feel like the administration had laid down these rules, but they haven't communicated all of these to every consultant.”
	2.3 Effects on Mental Health	**Fourth Year, MBBS student, 24 y Female:** We have, after coming back on campus we have tried and tested different ways to adjust, to this new sort of lifestyle in which night time is actually a predominant and its much safer and less anxiety driven time of the day to be wandering around campus' **Third Year, MBBS Student, 22 y, Female:** So, preventing the hostelites from visiting family on weekends does more harm in terms of mental health than just breaking the rules as opposed to genuinely restricting.
3. Sehat check application	3.1 Strengths	**Third Year, MBBS Student, 21 y, Male:** “I think it's brilliant. It's good. It's an innovation and it's a step forward in reducing the manpower and the man hours required to screen everyone.”
	3.2 Limitations	**Fourth Year, BSCN Student, 22 y, Female:** “First year students mistakenly marked ‘yes' on symptoms (and) as a result they were quarantined for 14 days.”
	3.3 Reasons for compliance	**Third Year, BSCN Student, 21 y, Female:** “It is mandatory to (fill it to) enter campus, so I fill it to make a pass for entrance.” **Third Year, MBBS Student, 21 y, Female:** “I think the app has generally just become a ticket to entering the courtyard”
	3.4 Reasons for lack of compliance	**Fourth Year, MBBS student, 24 y Female:** “You need internet access to go to Sehat App, and in most areas there is no internet there usually.”
	3.5 Recommendations for improvement	**Fifth Year, MBBS student, 24 y Male:** “I think there should be like a double check system put in place in the app, like ‘are you sure' before pressing enter.” **Fourth Year, MBBS student, 24 y Female:** “Maybe even like follow up on, get some information on our results because we could easily sign in and we could get our results.” **Third Year, MBBS Student, 21 y Female:** You can ask me what kind of cough, how many times did I have it? Five, six questions leading up from those generalized questions would just help in streamline who (should) get (the) COVID test.
4. Future recommendations	4.1 Targeted at juniors yet to return to campus	**Third year, MBBS Student, 21y Female**: “They should have dedicated learning spaces that are for them—to restrict them to their own batch, because those are the people that they're communicating with the most. For clinical years, we're dispersed throughout the hospital all day. But for them, they would be concentrated in the library or maybe more open spaces with easier distancing would be a better option as opposed to restricting them into their hostel rooms” **Fifth Year, MBBS Student, 24y Male:** “I would suggest restricting them to the university side, because there is really no need to take them to the hospital side. So they don't need to be exposed because the hospital already has a lot of suspected COVID patients”
	4.2 Targeted at leadership that strategizes and implements university policy	**Fifth Year, MBBS student, 24 y Male:** “I think mandatory testing should continue, it should be followed bi-weekly or once a month basis because there are so many asymptomatic infections, and we don't know the status.” **Fifth Year, MBBS student, 24 y Female:** “I would just like recommend more rigorous contact tracing and not associating shame to contact tracing, because a lot of people don't approach all the people they were in touch with because they are afraid, they might get them into trouble. Some sort of anonymous channel where they can safely report that they were exposed through a colleague or whatever, but I feel like that's effective because that's something I know is lacking right now.”

#### Effective safety interventions

Students appreciated certain safety interventions implemented by the university administration for their safe return to campus during the pandemic. Most students expressed agreement toward the weekly COVID-19 testing.

“*Mandatory COVID-19 testing for students was effective in picking up asymptomatic infections which would otherwise have been missed, hence stopping the source of transmission then and there*.” (5th year, MBBS student, 24 y Male)

Students mentioned that “… *because most people who were infected were (actually) asymptomatic so mandatory testing was most effective in limiting spread of infection*.” (3rd Year, BSCN student, 21 y Female)

With regards to subsequent effects of mask wearing policy, a student reported: “*the culture created on campus of mask wearing is great. (Hence) there is peer pressure associated for patients as well as attendings*.” (3rd Year, MBBS student, 22 y Male)

An important step in teaching was having classes in a hybrid online-onsite fashion. A student noted that, “*online sessions along with limiting the number of people in physical sessions has also been effective but online sessions are still more preferable*” (4th Year, MBBS student, 23y Female).

#### Safety interventions with limited effectiveness

The “Stay Strong” campaign was regarded as less effective intervention. Students claimed being well versed with SOPs regarding COVID-19 and wanted interventions translating into actions. One student stated that “*the stay strong campaign was initially good for motivation, however, there are better approaches that can be taken like opening up SRC*.” (5th Year, MBBS Student, 23y Male)

Some students also commented unfavorably on the restrictions placed on student spaces (specifically the policy to stay within academic bubbles after 11 p.m.). A student reported that “*we are not allowed to leave the hostels after 11 p.m. But the campus is empty at that time. The main exposure that we have is during the day, in our clinics*” (4th Year, MBBS Student, 23y Male).

#### Sehat check application

Students considered the application to be a good initiative. It prompted them to get the COVID-19 test done in case of entering relevant symptoms in the app. A student stated that “*It's a brilliant innovation and a step forward in reducing the manpower and man hours required to screen everyone manually*.” (3rd Year, MBBS Student, 21 male)

However, they also felt that improving the app would increase its usage and efficacy. A student suggested “*You can ask me what kind of cough and its frequency. Probes leading up from generalized questions would help streamline who (should) get (the) COVID test*.” (3rd Year, MBBS Student, 21 y, Female)

Students also stated that human errors while filling out application led to successive steps in the protocol that cannot be averted in time: “*Once I accidently clicked yes instead of no and it showed red, and I had multiple e-mails and calls*” (5th Year, MBBS Student, 24y old male). Another student recommended that “*there should be a double check system in the app, like ‘are you sure' before pressing enter*.” (5th Year, MBBS Student, 24y old male)

#### Future recommendations

Suggestions for bringing junior students back to campus mostly focused on strict adherence to SOPs. “*(Juniors) need to be respectful of the fact that hard work has been put into bringing and phasing in batches, so think of the community less than the individual*” (4th Year, MBBS, 23y old female).

Other ideas to inform policy for safe return to campus included initiating contact tracing systems and anonymous communication channels to report COVID-19 exposure or breach of relevant SOPs. A student proposed “*more rigorous contact tracing and not associating shame with it, because most students don't approach people, they were in touch with for fear of getting them into trouble. So, an anonymous channel for safely reporting exposure would be helpful*.” (5th Year, MBBS student, 24y female)

## Discussion

### Main findings

This study is the first account of the outcomes of 10 interventions targeting safe return of students back to campus at a large teaching hospital in Pakistan. These outcomes focus on the primary stakeholders and recipients of these interventions, students themselves, by shedding light on their perspectives around safety, usefulness of these interventions and behavior change. The results of this study center around four main findings: 1 surveys and FGDs were congruent in highlighting the three most successful interventions to be regular COVID testing, mandatory wearing of face masks at all times and a phased approach to bringing students back; 2 interventions perceived to be less effective being restrictions in student movement (both in terms of time and space), Town Hall meetings and the Stay Strong campaign; 3 need for bidirectional communication channels and feedback forums to help bolster and refine policy; and 4 feasibility of Sehat Check Application.

### Interventions with highest agreement

Frequent testing can help in timely detection of COVID-19 and curbing its spread (15). Studies have shown that regular, on-campus testing for COVID-19 is a highly favorable and adhered to prevention strategy among university students (31, 32). It is associated with lowered anxiety, feeling safe on campus and satisfaction toward university administration in taking effective steps to ensure student safety (31). This is in accordance with our findings which showed that students felt content after testing negative for the infection. It also helped them make more informed decisions about meeting people and engaging in activities. As global restrictions eased (33) presently, although frequent testing is less carried out, students are encouraged to get tested if they feel any symptoms similar to COVID-19. And this facility is easily accessible to students for all hours in the university.

Wearing face masks is important for preventing the spread of COVID-19 infection in the community. About 97% students in three medical universities in Karachi held positive attitudes toward wearing face masks as a protective measure against COVID-19 (34). A vast majority of students in our study also considered the mandatory policy of wearing face masks as a robust safety measure. Considering global restrictions are eased on wearing masks, sanitization, and social distancing (35), within the university academic settings it is no longer mandatory to do so, however for all clinical rotations' students are obliged to wear masks, continue sanitization and maintain social distancing.

As per directives of the Higher Education Pakistan (HEC), students whose learning depended on in-person training and learning were brought back on campus (36). These included the clinical batches of third, fourth and fifth year students. Using a staggering approach helped acquaint each batch with the new campus SOPs while giving them adequate time to adjust. A similar approach was adopted internationally in which positive outcomes of using a phased approach to resume on campus classes were reported (37, 38).

### Interventions that need reform

An emerging theme in FGDs was the unfavorable attitude of students residing in dormitories toward the curfew as they perceived on-campus exposure to be low during nighttime. Considering the university teaching hospital is attached, the curfew was there to ensure students do not leave the academic bubble and be exposed to the infection. In addition, it is more difficult to maintain infection control in hostels and any outbreaks can have serious consequences for all the residents (39). However, perhaps this was not clearly communicated to the students, and implies effective communication is needed between the leadership and students to resolve any conflicts or disagreements. In the present study, the curfew was removed once the Pakistan government lifted all COVID related restrictions (40).

In the present study, students did not favor both virtual or in person Town hall meetings held by leadership. These meetings were held to keep students informed of the actions taken by the university regarding their academics and general environment. Town hall meetings have been reported to increase students' awareness and knowledge regarding programme (41). However, students in the present study questioned its purpose. Although students were also encouraged to voice their concerns during these meetings, perhaps they felt it more as authoritarian, rather as a dialogue. Although in person town hall meetings have resumed after ease of restrictions (40), the findings of the present study imply that it's important to rethink the purpose of town hall meetings and clarify it from the perspective of students, for example to convey information, rules, and procedures, or as a means of engaging students in decision making.

With regards to “Stay Strong Campaign,” students claimed to be aware of the precautions related to COVID-19, hence they found pictorial displays of safety measures on campus to be of little value. Although this pictorial display discontinued once the pandemic eased (40), it implies as adult learners (42) students want to create meaningful interpretation of all activities. While initially this pictorial display was helpful, later it could have been replaced by a better alternative, for example, more interactive virtual content offering updated information would get better engagement or infographics depicting a selection of topics from the social and behavioral sciences relevant during a pandemic (43).

### Need for communication and feedback

In the present study, students preferred to be involved in formulating policies that affected them. Presently student representation is on certain academic committees such as research and curriculum, perhaps it's time to consider how best to further engage them in academic and health policies and procedures considering their opinions will enable the administration to foresee and maximize policy impact along with ensuring stronger student compliance. As part of bidirectional communication, regular student feedback could help in giving them a sense of ownership and motivation to adhere to safety protocols. Evidence also indicates that engaging students in medical education can promote better academic and health outcomes (44, 45).

In the present study, students were not in favor of the Sehat check application. For the present study, the intent of the Sehat check application was purely observational—that is disease surveillance and limiting infection spread by informing the regulating bodies of student infection rate for preventive measures. Although the idea of a screening application was appreciated and successful in this aspect, some technical limitations hindered its potential. Numerous health applications have been developed globally in response to COVID-19 (37, 46–48). Most commonly reported limitation of these applications was lack of methodological rigor (49). In the present study students' suggestions for improving the application included asking comprehensive questions for symptom detection, features for contact tracing, and providing updated information regarding pandemic globally. Incorporating these changes can refine the application to better achieve its objective. However, following ease of restrictions (40), the screening application was discontinued and the university plans to refine it for better usage and outcomes.

### Strengths

This study employed a carefully curated, multifaceted, approach to evaluate the efficacy of policies instituted and explore challenges faced by students upon returning to campus. A thorough literature search made it evident that there is a relative dearth of similar literature in the region and in developing countries. Moreover, while significant strides have been made in understanding policy experts' perspectives, a concerted effort is needed to delve into the primary stakeholders and recipients of these targeted policies -the students. Our study maintains these vital perspectives at the forefront, making it one of the first endeavors from a resource-limited setting. Our rigorous de-identification processes ensured that the feedback was anonymous, and protected. By amassing perspectives on the ability of each intervention to provide a safe environment and its effect on student behavior (self-reflected), we got valuable insights on perceptions around safety and behavioral patterns. An unexpectedly high proportion of students participated in FGDs, which further elucidates the importance of hearing and sharing the recipient side of the story. Pre-interview training of the study leads allowed standardization among different groups thereby strengthening reliability of the results. Both the execution and the assessment of our screening mobile application shed light on the scope, experiences, and efficacy of technology-driven innovations in a developing country.

### Limitations

Although the target population was diverse and well-spread over five different academic years from two programs, our study was limited to a cross-sectional single setting design in an urban environment. There was a slightly suboptimal response rate (33%) which can be attributed to two possible reasons. First, with the major paradigm shift from conventional platforms to virtual ones during the pandemic, it is possible that there was an information overload on students' emails (announcements, teaching instruction and other research dissemination) leading to less responsiveness overall. Second, the survey was disseminated a few weeks after students had returned to campus, which also coincided with the time that they were catching up on the significant backlog because of the pandemic. This could have resulted in students having less time to enroll as volunteer participants in research projects. However, it is reported that response representativeness is more important than response rate in survey research and in the present study, the sample is representative of both the medical and nursing students (50). Also, the aim of the study was to explore the usefulness of these intervention to support students for their learning, and in addition to the survey based responses, focus group discussions allowed to explore in depth to address the research objectives.

### Future implications

This study demarcates that mandatory COVID-19 regular testing and wearing of facemasks were the most effective interventions in curbing the spread of pandemic. In resource and time limited settings, these two key strategies should be the focus of efforts and budgeting. We also found that bidirectional communication channels are fundamental and requisite during ever-changing policies and regulations. Where possible, this needs to also gather real-time anonymous feedback and identify key areas that need further promulgation and those that need to be replaced with more effective ones. Thus, for practitioners, it is paramount to establish mechanisms to clearly communicate, implement and evaluate strategies at regular intervals. Concurrently, engaging student representatives in decision making or implementation processes (such as “pilot” before “roll-out”) would allow any potential issues to be managed early on.

## Conclusion

These findings can be used to conduct further research, after a follow-up period, to implement, evaluate and categorize policies into one of many such arms: those that have long-term sustainability, those that saw attenuation after an initial peak and those that improved over time (both in terms of safety of intervention and its effect on student behavior patterns). To ensure depth and a holistic understanding, gathering faculty perspectives on the same or similar policies could follow this process. Likewise, to augment the breath of perspectives, more work should ensue on institutions spread over diverse socioeconomic and cultural setups. Bidirectional communication channels are fundamental and requisite during ever-changing policies and regulations. Engaging student representatives in decision making or implementation processes (such as “pilot” before “roll-out”) would allow any potential issues to be managed early on. Gather real-time anonymous feedback and identify key areas that need further promulgation and those that need to be replaced with more effective ones.

## Practice points

1 Bidirectional communication channels are fundamental and requisite during ever-changing policies and regulations
2 Engaging student representatives in decision making or implementation processes (such as “pilot” before “roll-out”) would allow any potential issues to be managed early on.
3 Gather real-time anonymous feedback and identify key areas that need further promulgation and those that need to be replaced with more effective ones.
4 In resource and time limited settings. mandatory COVID-19 regular testing and wearing of facemasks were the most effective interventions in curbing the spread of pandemic.

## Data availability statement

The original contributions presented in the study are included in the article/supplementary material, further inquiries can be directed to the corresponding author.

## Ethics statement

The studies involving human participants were reviewed and approved by Ethical Review Committee (ERC) Aga Khan University. The patients/participants provided their written informed consent to participate in this study.

## Author contributions

SM, SI, AN, RK, UI, and MT contributed to the conception and design of the study. HS, AH, NA, AJ, SZ, and KA contributed to the acquisition and interpretation of the data. All authors contributed to the write-up of early versions of the manuscript, agree accountability for the accuracy and integrity of the work and approved the final version of the submitted manuscript.

## Conflict of interest

The authors declare that the research was conducted in the absence of any commercial or financial relationships that could be construed as a potential conflict of interest.

## Publisher's note

All claims expressed in this article are solely those of the authors and do not necessarily represent those of their affiliated organizations, or those of the publisher, the editors and the reviewers. Any product that may be evaluated in this article, or claim that may be made by its manufacturer, is not guaranteed or endorsed by the publisher.
